# Nanoporous thin films in optical waveguide spectroscopy for chemical analytics

**DOI:** 10.1007/s00216-020-02452-8

**Published:** 2020-02-27

**Authors:** Wolfgang Knoll, Omar Azzaroni, Hatice Duran, Julia Kunze-Liebhäuser, King Hang Aaron Lau, Erik Reimhult, Basit Yameen

**Affiliations:** 1 Competence Centre for Electrochemical Surface Technology, 2700 Wiener Neustadt, Austria; 2 AIT Austrian Institute of Technology GmbH, 3430 Tulln an der Donau, Austria; 3 Instituto de Investigaciones Fisicoquímicas Teóricas y Aplicadas, Departamento de Química, Facultad de Ciencias Exactas, Universidad Nacional de LaPlata – CONICET, 1900 La Plata, Argentina; 4 Department of Materials Science and Nanotechnology Engineering, TOBB University of Economics and Technology, 06560 Ankara, Turkey; 5 Institute for Physical Chemistry, Leopold-Franzens-Universität Innsbruck, 6020 Innsbruck, Austria; 6 Department of Pure and Applied Chemistry, University of Strathclyde, Glasgow, G1 1XL UK; 7 Department of Nanobiotechnology, University of Natural Resources and Life Sciences, 1190 Vienna, Austria; 8 Department of Chemistry and Chemical Engineering, Syed Babar Ali School of Science and Engineering, Lahore University of Management Sciences, Lahore, 54762 Pakistan

**Keywords:** Anodization, Colloid lithography, e-Beam lithography, Nanoporous thin films, Optical waveguide spectroscopy, Chemical and biosensing, Polymer nanorod array

## Abstract

Spectroscopy with planar optical waveguides is still an active field of research for the quantitative analysis of various supramolecular surface architectures and processes, and for applications in integrated optical chip communication, direct chemical sensing, etc. In this contribution, we summarize some recent development in optical waveguide spectroscopy using nanoporous thin films as the planar substrates that can guide the light just as well as bulk thin films. This is because the nanoporosity is at a spacial length-scale that is far below the wavelength of the guided light; hence, it does not lead to an enhanced scattering or additional losses of the optical guided modes. The pores have mainly two effects: they generate an enormous inner surface (up to a factor of 100 higher than the mere geometric dimensions of the planar substrate) and they allow for the exchange of material and charges between the two sides of the solid thin film. We demonstrate this for several different scenarios including anodized aluminum oxide layers for the ultrasensitive determination of the refractive index of fluids, or the label-free detection of small analytes binding from the pore inner volume to receptors immobilized on the pore surface. Using a thin film of Ti metal for the anodization results in a nanotube array offering an even further enhanced inner surface and the possibility to apply electrical potentials via the resulting TiO_2_ semiconducting waveguide structure. Nanoporous substrates fabricated from SiN_*x*_ thin films by colloid lithography, or made from SiO_2_ by e-beam lithography, will be presented as examples where the porosity is used to allow for the passage of ions in the case of tethered lipid bilayer membranes fused on top of the light-guiding layer, or the transport of protons through membranes used in fuel cell applications. The final example that we present concerns the replication of the nanopore structure by polymers in a process that leads to a nanorod array that is equally well suited to guide the light as the mold; however, it opens a totally new field for integrated optics formats for direct chemical and biomedical sensing with an extension to even molecularly imprinted structures.

**Figure:**
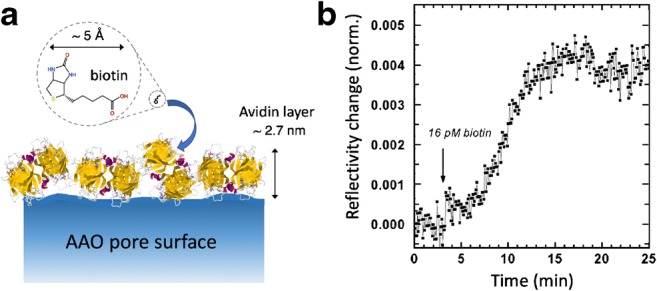
Graphical abstract

Graphical abstract

## Introduction

Dielectric waveguide structures, either as optical fibers or in planar or strip waveguide formats, that are able to guide light in defined modes have been around for many decades [1–3]. However, they still attract significant attention [4], mostly for various applications, notably for chemical and biomedical sensing [5–8].

Whether implemented as a linear guide [9], e.g., coupled to a microfluidic chip, or in more sophisticated designs like in integrated Mach–Zehnder interferometers [10, 11], coupled to a ring resonator [12], or in full chip-scale integration [13], in almost all of these cases the light propagates nearly completely within the waveguide structure. Only the evanescent part of the optical field senses the proximity of the waveguide in the several hundred nanometers to micron range [14]. Hence, any recognition and binding event of an analyte molecule from solution to the (functionalized) surface of the waveguide structure modifies the whole optical (refractive index) configuration only a little bit. The consequence is that the energy–momentum relation (dispersion) of the guided mode changes only slightly, as seen then by only a minute shift of the coupling angle in a prism or grating coupling format [15, 16] (or in a wavelength shift of the resonance in broadband excitation [16]).

**Fig. 1:**
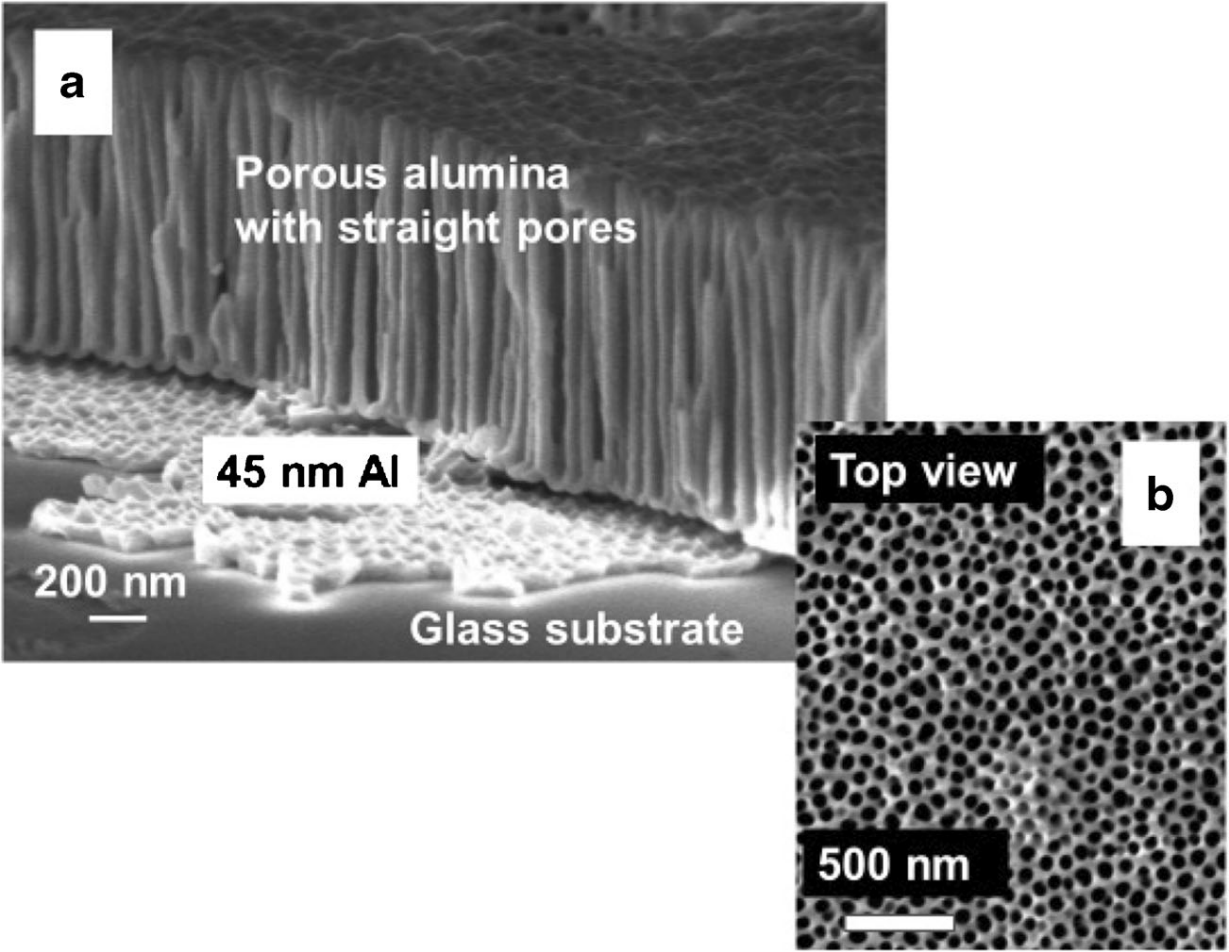
Scanning electron microscopy (SEM) image of an anodized aluminum oxide (AAO) waveguide structure.** a** Partly broken film that shows the porous alumina layer with the straight nanopores reaching from the top surface to the bottom, the thin Al film that is left by not fully completing the anodization of the sputtered metal film, acting as the coupling gap in the waveguide spectroscopy, and parts of the glass substrate.** b** Top view of the nanoporous layer, showing the openings of the nanopores with a mean cross section that is substantially smaller than the wavelength of the guided light

In order to overcome this disadvantage and to enhance the sensitivity of optical waveguide platforms for sensing chemical or biomedical analyte molecules, we started experimenting with nanoporous thin films as waveguides [17, 18]. These structures (e.g., Fig. 1) generate an enormous inner surface that can be again functionalized, e.g., by capture molecules like receptors, yet they allow for the use of the (nearly) complete optical field of the guided mode for the sensing of the analyte [18, 19]. When properly designed, these structures guide the light just as well (i.e., without additional scattering losses by any refractive index heterogeneity) as do bulk optical waveguides.

While the first structures were based on anodized aluminum oxide (AAO) layers, a number of other thin film materials and structures have been reported, all showing the same combination of a (relatively) high refractive index dielectric thin film with a nanoporous inner architecture at dimensions that do not scatter the guided modes. Furthermore, these structures allow for the (nearly) free passage of the analyte molecules from the superstrate phase into the pores and to the pore walls where they can bind to the immobilized specific receptor units.

In the following we (i) summarize some of these novel materials that offer the potential to be used as waveguides, all with a huge inner surface that dramatically enhances the sensitivity as a sensor platform, (ii) demonstrate for some of them their use in an optical waveguide spectroscopic setup, and (iii) introduce various forms for their surface functionalization. This demonstrates the versatility of the concept for various fields of application in chemical analytics from biosensing to electrochemical diagnostics and from membrane biophysics to fuel cell research.

## Materials and methods

**Fig. 2:**
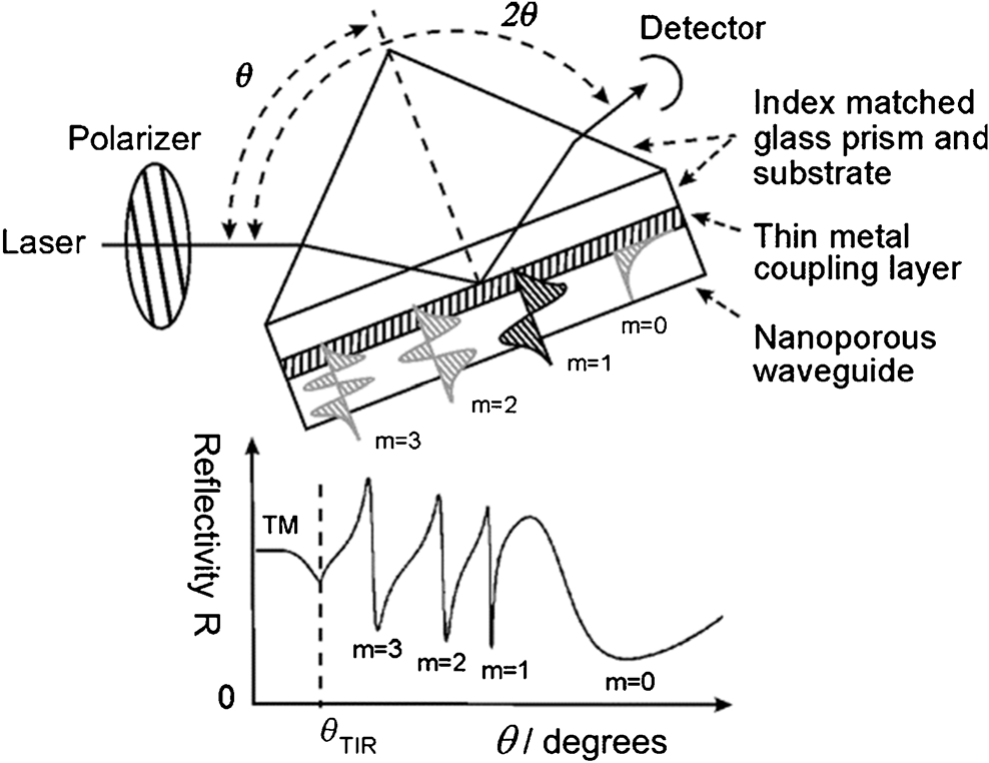
Schematics of the experimental setup used to monitor the guided optical modes. Upper part: a polarized laser is coupled via the prism and the metal layer to the dielectric (nanoporous) waveguide layer at an angle θ with the reflectivity detected at 2θ. In an angle scan measurement, the reflectivity is measured as the prism is rotated over a range of θ. Lower part: typical reflectivity as a function of θ, simulated assuming a high index prism and (index matched) glass substrate (LaSFN9), an aluminum coupling layer of 40 nm in thickness, and a nanoporous AAO waveguide layer placed in air. Different combinations of incidence laser wavelength, layer material, and thickness would change the details of the trace but the overall excitation of multiple sharp waveguide modes at specific θ would be retained

The concept of using (nanoporous) thin films as optical waveguides is schematically depicted in Fig. 2, upper part. A polarized laser beam is coupled via the prism and the metal layer to the dielectric (nanoporous) waveguide layer at an angle θ with the reflected light being detected at 2θ. At particular angles that fulfill the condition for energy and momentum matching between the incoming photons and the guided modes [20] various resonances can be identified in the reflectivity spectrum (lower part of Fig. 2). These represent the corresponding eigenmodes of the guided light, with an intensity distribution within the film schematically indicated and indexed according the order of the modes. Since the simulation was done with p-(i.e., transverse magnetic, TM)-polarized light the* m* = 0 mode corresponds to a surface plasmon mode propagating at the metal–dielectric interface [21].

Any change of the refractive index configuration of the substrate, of the guiding layer, or of the superstrate will result in a modification of the dispersion of the guided modes which will show up as a change of the angular position of the eigenmode resonances. Typically, the refractive index of the substrate and the metal coupling gap (at fixed temperature and pressure) are constant; any shift of the angular position of the resonances hence can be modeled as being caused by a change of the superstrate and the waveguide. Since the solid part, i.e., Al_2_O_3_, the SiO_2_, or the TiO_2_, can also be assumed to be constant, the only free parameter in the simulation of the spectra is the refractive index of the solvent of the superstrate and within the pores, as well as any coating on the pore walls that would also lead to a mode shift. And since we are dealing with a very high inner surface area, these shifts can be rather substantial, even for very thin coating (see below).

In order to complement the summary of nanoporous thin layers that are suited for this waveguide approach we also introduce a few examples with other materials, although we only present the corresponding waveguide spectroscopic characterization for some of them.

These thin films include TiO_2_ nanotube arrays that are also fabricated by anodization of metallic titanium [22], films from SiN_*x*_O_*y*_, nanostructured by colloidal lithography [23, 24], and self-standing porous silicon membranes produced by a photoelectrochemical etching process with highly ordered vertically aligned monodisperse channels [25].

## Results and discussion

### Waveguide spectroscopy for refractive index determination

The first example that we describe for the use of nanoporous waveguide structures concerns the determination of the refractive index of a liquid. As mentioned above, for a constant optical architecture with the substrate and the waveguide with its fixed thickness and porosity, the only free parameter that determines the angular position of the various mode resonances is the superstrate that also fills the pores. For a waveguide with unknown porosity, a first set of measurements with air or a liquid with a precisely known refractive index allows for the determination of this parameter, i.e., the fraction of the dielectric solid material, relative to the pore volume.

**Fig. 3:**
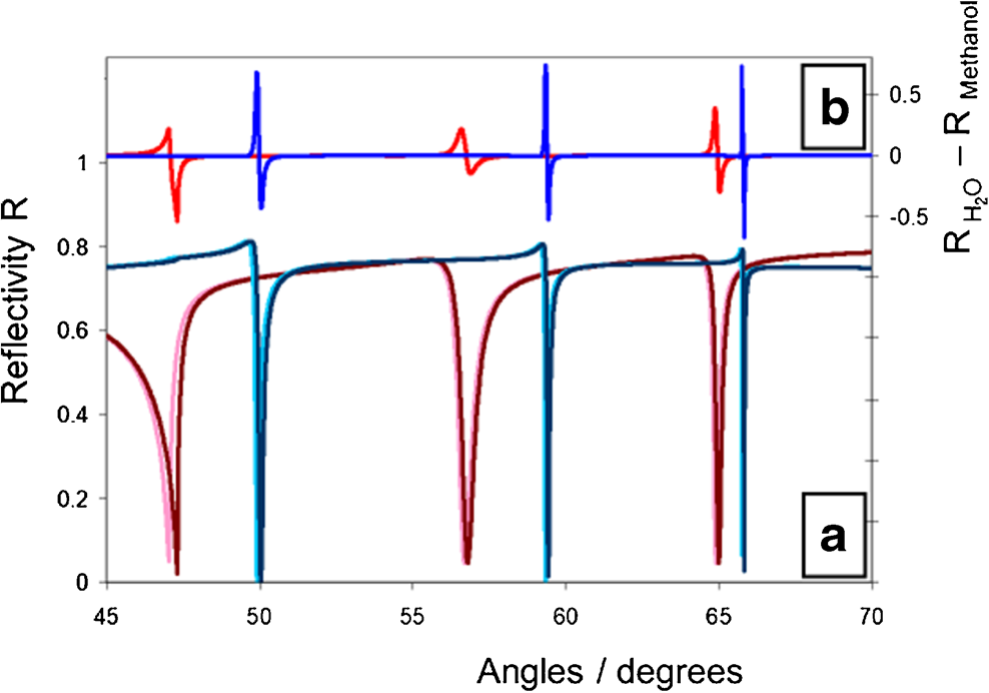
**a** Waveguide spectra, simulated for a 2-μm-thin nanoporous Al_2_O_3_ waveguide with an assumed porosity of 23% and a 45-nm-thick Au coupling layer, and the following refractive index values:* n*_Al2O3_ = 2.6,* n*_water_ = 1.3332,* n*_methanol_ = 1.3296; the blue spectra are simulated with s-polarized light, the red ones for p-polarization; light colors are for methanol, dark colors for water.** b** Difference spectra between water and methanol for the various modes

We demonstrate the sensitivity for such an experimental approach for the determination of the refractive index of an unknown liquid by simulating the change of the mode spectrum in going from water as the pore-filling superstrate to methanol. The refractive indices of the two liquids are rather similar: we assumed in the simulation for water,* n*_H2O_= 1.3332; and for methanol,* n*_methanol_ = 1.3296. The two sets of waveguide modes, for both liquids simulated with p- as well as with s-polarized light, are shown in Fig. 3a. The difference spectra shown in Fig. 3b demonstrate the high sensitivity of the method: the minor differences of the refractive index of water and methanol result in a change of reflectivity in the most sensitive mode of more than 50%!

### Waveguide spectroscopy for small analyte sensing

Application of nanoporous waveguide spectroscopy to the sensing of a low molecular weight small analyte molecule—biotin in our case (244.3 g/mol)—on the pore surfaces of AAO illustrates one of the advantages of the approach. High sensitivity is achieved owing to the very sharp resonances of waveguide modes intrinsic to the physical phenomenon of optical waveguiding [20], and to locating the surface binding reactions of interest within the nanoporous structure where the optical field is most intense and where there is a vast internal surface area for the reactions to occur [19, 26]. In the case of a typical 1-μm-thick AAO waveguide layer with pores 60 nm in diameter and arranged with ca. 100 nm center-to-center spacing between pores (cf. Fig. 1b), the internal surface area is 22 times that of the top flat surface. Therefore, even binding of a minute quantity of unlabeled material, when summed over the pore surfaces, may generate a significant change in the overall optical density and hence a detectable signal.

**Fig. 4:**
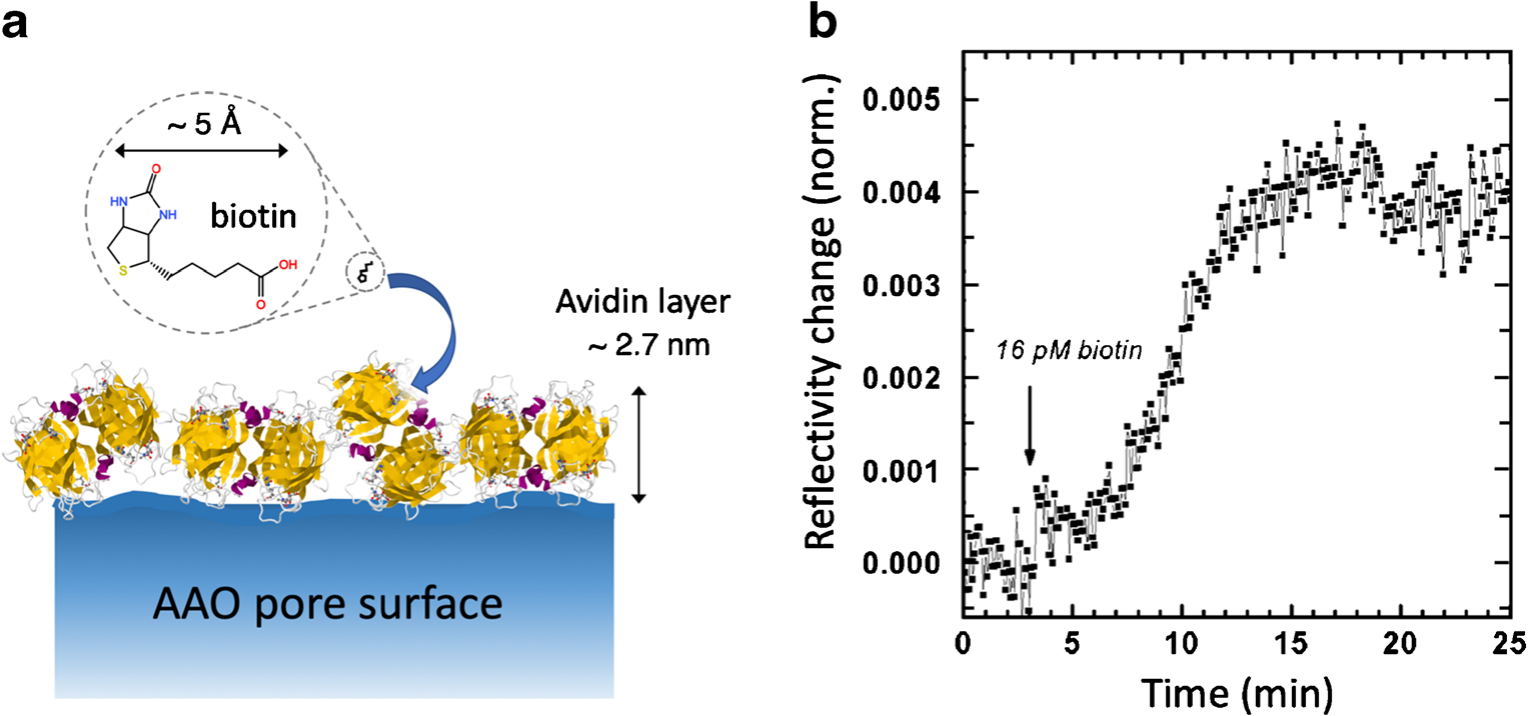
**a** Schematic of the surface binding of biotin onto a layer of avidin protein coating the pore surface of AAO, indicating the dimensions of the molecules.** b** The reflectivity change measured at the shoulder of a TE_0_ mode (minimum at 64.91°) due to the binding of 16 pM biotin in pH 7.4 phosphate buffered saline (PBS) to a monolayer of avidin. The reflectivity change was normalized to the original incident laser intensity estimated in Fresnel equation fitting of the angle scan. The avidin layer adsorbed on AAO was 2.7 nm thick. The pore diameter was 60 nm and the AAO thickness was ca. 1 μm. The data shown correspond to [26]

For the demonstration of detecting biotin binding, we prepared AAO functionalized with avidin (Fig. 4a). Avidin is a glycoprotein with a strong non-covalent interaction with biotin (*K*_a_ = 10^15^ M^−1^). The protein has two pairs of binding pockets for biotin, a pair on its top and another on its bottom face. Thus, surface-bound avidin may further bind with free biotin in solution for sensing purposes regardless of the protein’s surface orientation. Figure 4b shows the kinetics of binding biotin from a 16-pM solution, measured with the sharpest s-polarization (i.e., transverse electric, TE) fundamental waveguide mode (TE_0_). At this concentration, there is only one biotin per 100 μm^3^ of buffer (i.e., 10^−15^ mL). Since the volume of each 60-nm-diameter, 1-μm-long pore is only 3 × 10^−3^ μm^3^, there was much fewer than one molecule per pore. The slow rise in reflectivity due to biotin binding therefore represented the rate of molecular transport in sensing (flow combined with diffusion into the pores) at low concentrations. The final reflectivity change after 10 min was *ΔR* = 0.004, which corresponded to a ca. 0.004° angle shift of the TE_0_ mode in this particular AAO sample, and an effective increase of ca. 0.007 nm in the average thickness of the surface molecular layer estimated from effective medium theory calculations (assuming refractive index = 1.5) [26].

### Monitoring buildup of supramolecular architectures

**Fig. 5:**
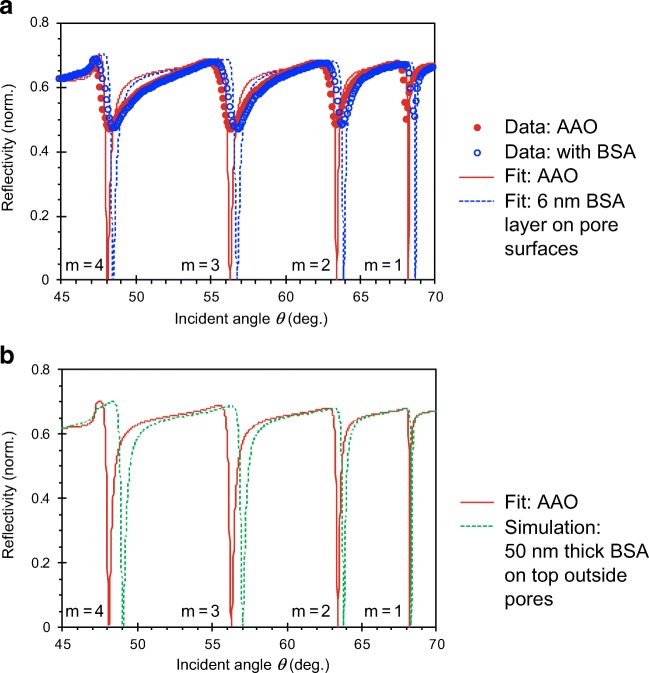
**a** Reflectivity angle scans of an AAO waveguide before (filled red circles) and after BSA adsorption (open blue circles) on the pore surfaces from a solution of 50 mM BSA in PBS held at pH 5.0 (BSA’s isoelectric point). The pore diameter of this AAO was ca. 31 nm. The lines traces are fittings from Fresnel calculations.** b** Fresnel calculations of the theoretical reflectivity angle scans of the bare AAO (same as in** A**) compared with that due to the addition of a hypothetical 50-nm-thick BSA layer on top of the AAO. The data shown are from [18]. Note that for this structure we used a Au coupling layer instead of Al; the laser wavelength was λ = 633 nm

Nanoporous waveguide spectroscopy can certainly also be applied to characterizing the surface binding of larger (bio)macromolecules. In a first demonstration [19], bovine serum albumin (BSA) dissolved in PBS was physisorbed onto AAO to cause angle shifts in the waveguide modes. Figure 5a shows the reflectivity vs. angle scan of the first four p-polarization (i.e., TM) modes labelled* m* = 1 to 4. Of particular note was that all the waveguide modes shifted by approximately the same amount. As discussed above, cf. Fig. 2, the different waveguide modes have different spatial distributions in field intensities through the thickness of the nanoporous waveguide layer. Individual modes are most sensitive to changes occurring where the amplitudes are highest. Therefore, parallel shifts indicated that BSA had diffused efficiently down the pores and adsorbed uniformly on the pore surfaces.

The easily measurable large angle shifts shown in Fig. 5a also reflected the high sensitivity towards surface processes. BSA is a protein ca. 8 × 7 × 6 nm^3^ in size [27], and the ca. 0.5° angle shift measured for TM_1_ reflected the adsorption of a ca. 6-nm-thick layer (i.e., an angle shift of 0.08°/nm thickness change), as estimated from effective medium theory calculations [19]. Since ca. 0.001° angle shifts may be measured with commercially available goniometers, an adlayer thickness sensitivity of ca. 0.01 nm should be routinely possible, i.e., in the same range demonstrated in Fig. 4 for biotin binding.

The high detection sensitivity possible with nanoporous waveguide spectroscopy can also be seen in a theoretical comparison with conventional flat surface waveguide sensing. Figure 5b compares the waveguide response calculated from Fresnel equations for the bare AAO waveguide with the theoretical response if an implausibly 50-nm-thick BSA layer were adsorbed on top of the waveguide. The simulated TM_1_ angle change was only ca. 0.1° (i.e., 0.002°/nm) and that for TM_4_ was ca. 1.1° (i.e., 0.022°/nm), both of which are smaller than measured for actual BSA adsorption on the pore walls (Fig. 5a). This simulation illustrates two points of interest: (1) a much smaller angle response would result for conventional waveguide spectroscopy that relied on sensing of surface processes only on the top surface of a waveguide, and (2) waveguide spectroscopy could distinguish between surface processes inside and on top of the nanoporous layer, because more of the guided field would be “leaked” out of the waveguide for a higher-order mode and be more sensitive to processes outside of the nanopores. Conversely, lower-order modes are more sensitive to internal pore surface processes.

**Fig. 6:**
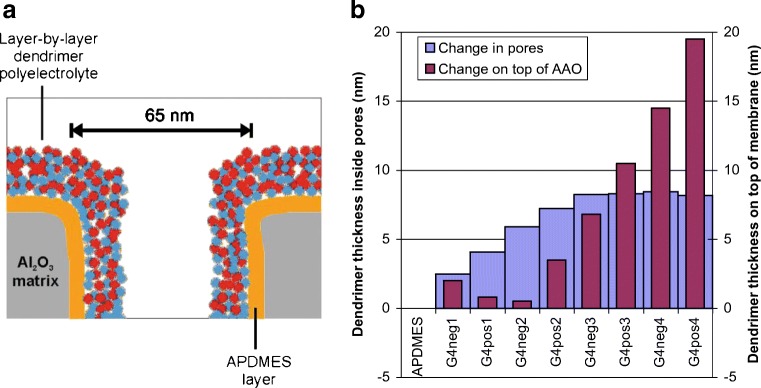
**a** Schematic of multiple dendritic polyelectrolyte layers deposited on AAO functionalized with 3-aminopropyl(diethoxy)methylsilane (APDMES) to give an initial positive surface charge. Note the additional layers deposited on the top vs. the interior side pore walls.** b** Waveguide measurements of the layer thickness from LbL deposition of 4th-generation dendritic polyelectrolytes with hydrazine cores (G4neg and G4pos for cationic and anionic dendrimer surface charges, respectively). The initial pore diameter was 65 nm and the dendrimers were dissolved in pH 7.4 PBS at 1 mg/mL. The data shown correspond to [28]

This differential sensitivity of different modes for inside and outside of the nanopores was exploited in a series of studies for characterizing the layer-by-layer (LbL) deposition of polyelectrolytes and proteins in AAO [28, 29]. Figure 6a shows a schematic of the surface layer structure after successive LbL steps. In this example, a pair of 4th-generation polyelectrolyte dendrimers with a ca. 7 nm diameter and cationic (“G4pos”) or anionic (“G4neg”) dendrimer surface terminations was used. Although such an LbL polyelectrolyte deposition is routine on a flat surface, the situation is more complex in a nanoporous system. This is because the polyelectrolyte must first diffuse into the nanopore before deposition may take place, but there may be a physical size constraint for pore entry. Furthermore, pore entry may be further limited by electrostatic repulsion between incoming polyelectrolytes and the same molecules that have already attached around the pore entrances during any given deposition step.

This is exactly our observation, as Fig. 6b shows that the thickness change in the pores essentially stopped after deposition of the second bilayer in 65-nm-wide AAO nanopores, but the deposition on the top surface of the AAO membrane continued in a linear fashion [28]. In fact, analogous results were obtained for the LbL deposition of avidin and BSA carrying opposite net charges [29]. Interestingly, in both dendrimer and protein LbL deposition, internal pore deposition effectively ceased when the open pore diameter estimated from effective medium theory was still greater than three times the diameter of the polyelectrolyte, which indicated a strong electrostatic effect for pore entry. This was the case even at very high buffer salt concentrations (0.5 M NaCl) when the electrostatic screening length should be significantly shorter than the size of the polyelectrolytes. At lower salt concentrations, the effect was even stronger and may be further exploited for the differential functionalization of nanoporous membranes inside and outside of the pores [28].

### TiO_2_ nanotube waveguides

Self-organized, anodic TiO_2_ nanotube (NT) arrays offer a large spectrum of possible applications [22]. Among the most important optical implementations is their use as photonic crystals in fiber lasers and demultiplexers, and as nanostructured electrodes for surface enhanced Raman spectroscopy [30, 31]. Independent of the chemical surroundings, optical field enhancement can occur as a result of the presence of additional free electrons in TiO_2_ [31]. Charge transfer processes and photon scattering are enhanced through the directionality of incoming light which is triggered through the nanotube morphology [32]. The collective optical modes in the lattice structure cause high sensitivity in these photonic crystals despite the presence of defects.

Self-organized, anodic TiO_2_ NTs are synthesized through anodic oxidation of titanium (Ti) metal in fluoride-containing electrolytes [22]. Depending on the electrochemical conditions, their length varies between 0.3 and 260 μm, and their diameter can range from 30 to 200 nm [33]. Zwilling et al. [34] were the first to report on anodic nanotube formation in aqueous, fluoride-containing electrolyte. Later, extensive variation of the growth conditions allowed for optimization of the faradaic efficiency of the process and in turn for aspect ratio increase [35–37], and a large number of NT geometries and types became accessible [22].

**Fig. 7:**
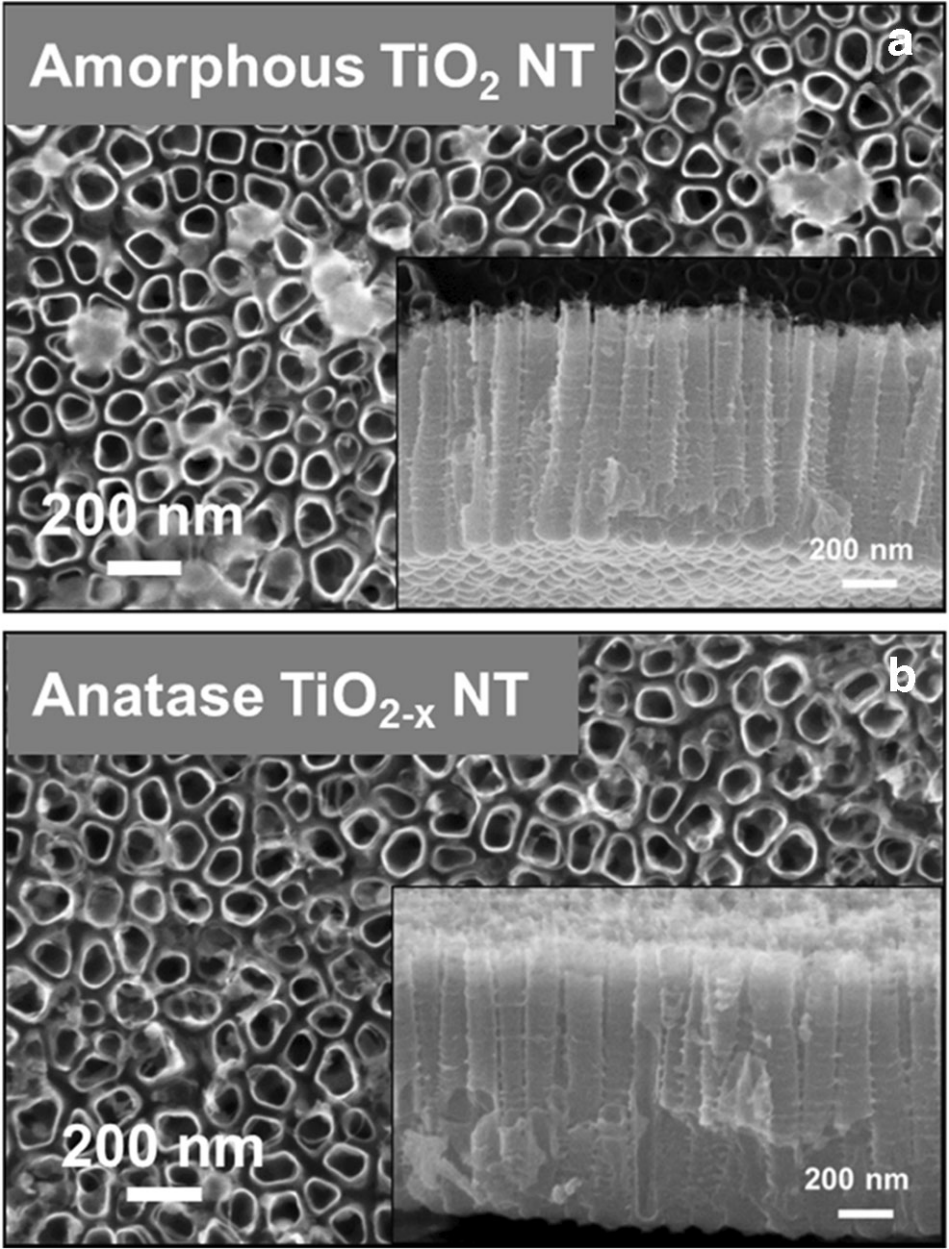
SEM images of top and cross sections of** a** amorphous TiO_2_ NTs and** b** anatase TiO_2−*x*_ NTs. Reproduced with permission from Ref. [46]. © 2017 Elsevier B.V. All rights reserved

The formation mechanism of self-organized anodic TiO_2_ NTs comprises the formation of breakdown sites in the barrier oxide layer and, after an initiation phase, the growth of highly regular parallel NTs [38, 39]. During the growth, fluoride species accumulate at the oxide–metal interface [40], and stable NT formation has now been explained through a combination of plastic oxide flow [41] and field-assisted dissolution processes [35, 42]. TiO_2_ NTs can be grown on sputter-deposited Ti metal [43], which is beneficial for their use as waveguides. As-grown TiO_2_ NTs are amorphous and they can be thermally converted to anatase (Fig. 7). Oxygen-deficient anatase TiO_2−*x*_ (*x* < 2) NTs are produced by annealing at 400 °C under argon (Ar) atmosphere [44], which leads to the presence of oxygen vacancies and enhanced charge-transfer properties.

The crystallographic structure (amorphous, anatase, rutile, and brookite) determines all electronic, ionic, and optical properties of TiO_2_ NTs. Anatase shows the highest electron mobility [45] and is therefore often used in applications where electron conduction is required. The optical band gap of anatase and rutile is 3.2 and 3.0 eV, respectively. TiO_2_ is applicable in a wide range of optical applications owing to its high refractive index.

### Nanoporous substrates, fabricated by colloid lithography, for buildup of supported lipid bilayer membranes

**Fig. 8:**
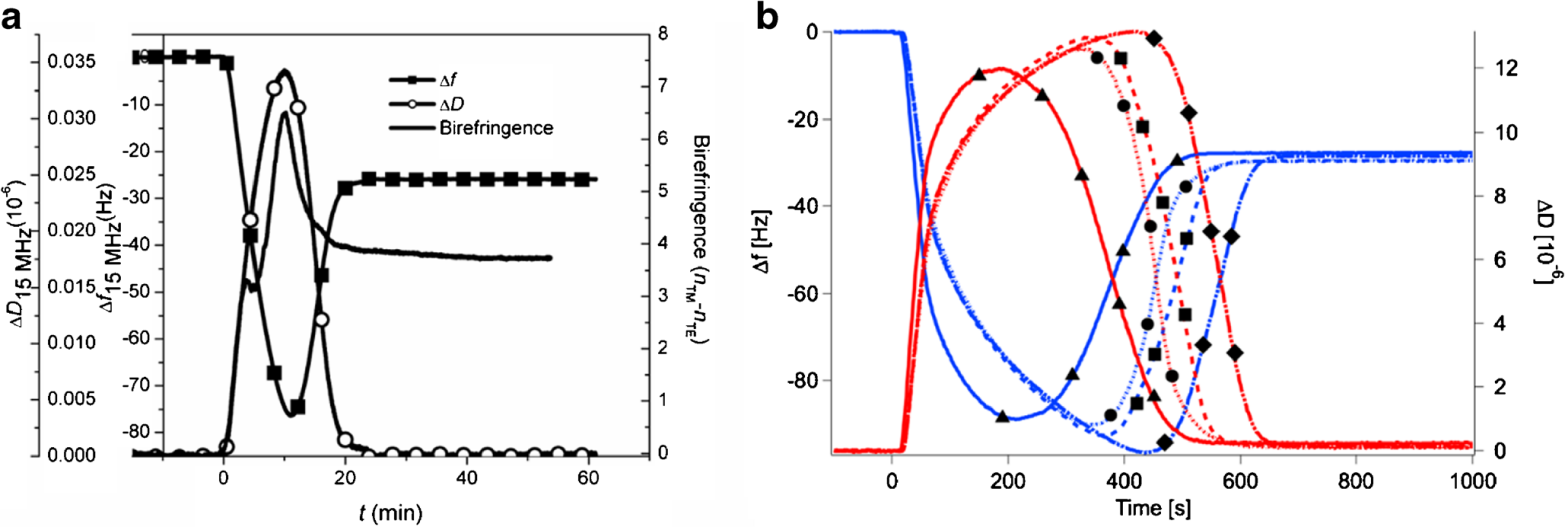
**a** Comparison of quartz crystal with dissipation (QCM-D) monitoring and dual polarization interferometry birefringence analysis for self-assembly of a supported lipid bilayer by liposome adsorption and rupture [51]. The peak in birefringence coincides closely with the peak in dissipation from QCM-D obtained under the same flow conditions. The dissipation is known to peak at the maximum concentration of liposomes on the surface [52].** b** QCM-D graph of supported lipid bilayer formation on unpatterned (triangles) and nanopore-patterned (pore diameter 40 nm [circles], 100 nm [squares], and 200 nm [diamonds]) oxidized silicon nitride. The final values of Δ*f* (ca. −26 Hz) and Δ*D* (ca. 0.2 × 10^−6^) are similar for all substrates [53]

Waveguide-based sensing was quickly adopted as a very sensitive method to study biomolecule adsorption on surfaces that could surpass the limit of detection of surface plasmon resonance (SPR) [47, 48]. Nanostructures have been shown to increase the sensitivity of waveguides to biomolecule adsorption even further [49]. Additionally, utilizing that multiple waveguide modes can propagate and sense over a large area, waveguide spectroscopy has also been used to measure conformational changes in adsorbed biomolecular layers [47]. As an important example, lipid membranes have high internal order and asymmetric optical properties. This has been exploited in waveguide spectroscopy by recording the difference in optical response from two orthogonal waveguide modes and this was used to analyze the deformation state of adsorbed liposomes [50] and the temporal distribution of liposomes and planar supported lipid bilayer during the vesicle rupture and supported membrane formation process (Fig. 8a) as well as the phase state of the supported membrane [51]. Thereby, detailed insights into the strength of the interaction between lipid membranes and the substrate as well as how membranes propagate on the surface could be gained.

Porous waveguides add a dimension to measurements since the waveguide modes respond to molecules adsorbing into pores and on the surface differently (see above). The development of such waveguide sensors with straight-etched pores in the 100-nm range is particularly interesting for the development of tools for model membrane research and for sensors using membranes as the sensor element. The first reason is that while most biosensor platforms use solid substrates that do not allow for sufficient space to incorporate transmembrane proteins into a membrane on the surface, the aperture and the aqueous volume provided by a pore make such integration possible. The second reason is that nanopores can connect to an electrode on the proximal side of the dielectric waveguide material and thereby enable electrochemical measurements across a supported lipid membrane spanning the pores. Thus, SPR-coupled waveguides that use a gold film to excite waveguide modes can be used as a dual-modality sensor system for supported, pore-spanning, model membranes.

Pore-spanning membranes have been used for electrochemical characterization of lipid membranes and membrane proteins for a long time [54]. However, such studies have mainly been limited to fragile and short-lived membranes spanning micromillimeter- and millimeter-sized apertures, which frequently produce measurements distorted by solvent residues from the painting of the membrane [54, 55]. Shrinking the aperture size and thereby the free-hanging membrane down to the submicrometer and preferably 100-nm range can significantly enhance the observed stability [55–57]. Assembly of lipid membranes has been achieved on substrates suitable for waveguides such as TiO_2_ [48, 58], SiO_2_ [52], and SiN_*x*_O_*y*_ [57]. Such dielectric materials can be patterned with nanopores of controlled size, shape, and density. The Steinem group has shown different approaches to how to self-assemble lipid membranes on porous anodized alumina and nanopore-patterned silica functionalized with alkane self-assembled monolayers (SAMs) [57, 59]. The parameters and probabilities that guide direct self-assembly of membrane-spanning pores in the 100-nm range by vesicle adsorption were also investigated on surface-oxidized silicon nitride thin films [53] suitable for waveguiding (Fig. 8b). These publications also demonstrate that an ideal combination for functional waveguide sensing on self-assembled lipid membranes would be a waveguide rich in silicon oxide with a relatively low density of nanopores for direct self-assembly of pore-spanning membranes from liposomes or nanopore substrates functionalized with a hydrophobic monolayer for assembling membranes with a tight seal. In both cases, an ideal pore geometry seems to be straight pores with sharp edges and apertures in the 100-nm range.

**Fig. 9:**
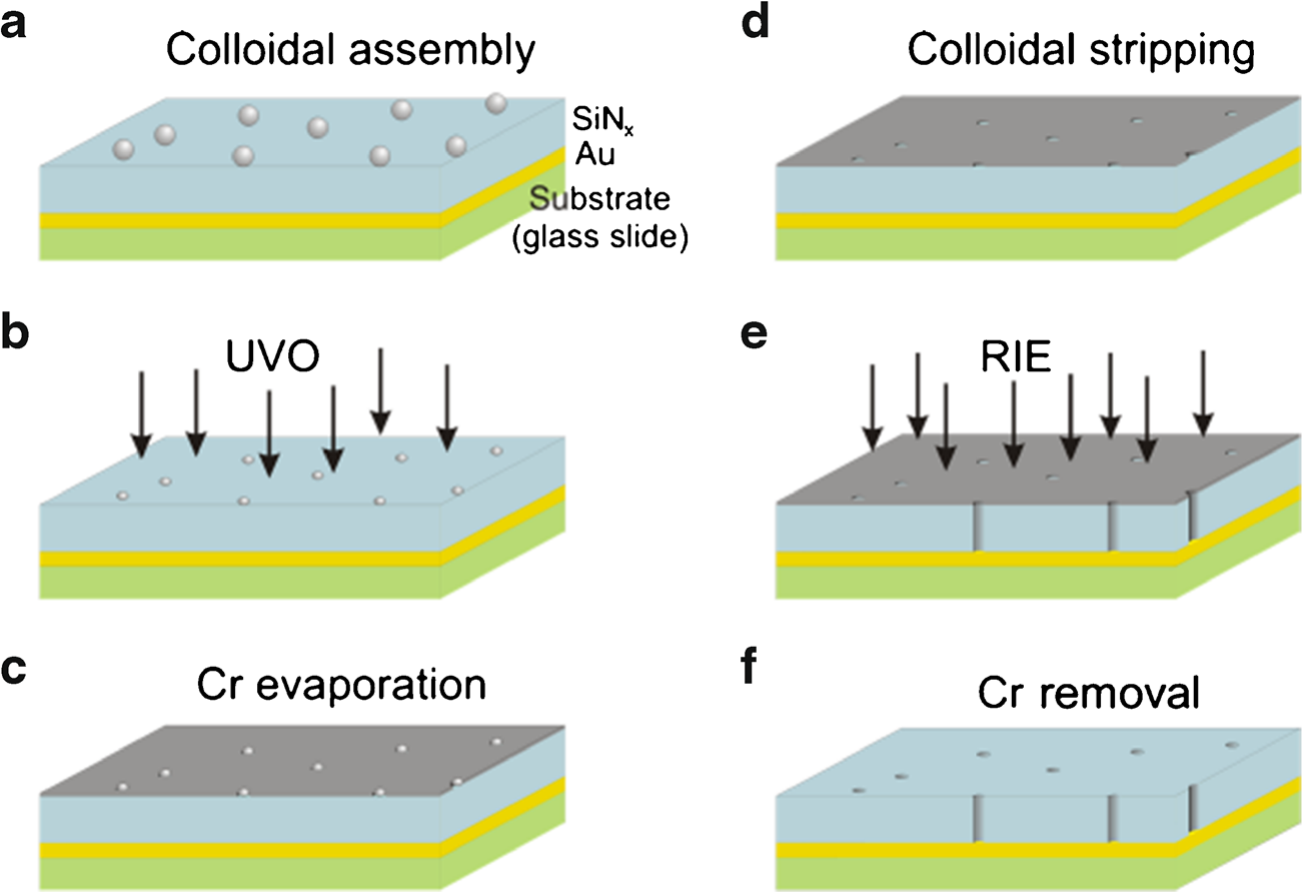
Schematics of the manufacturing process of a porous waveguide/nanoarray electrode.** a** A high refractive index glass slide is coated with a Au layer using a thin Ti layer for improved adhesion. On top of the Au layer, a dielectric film of desired thickness and refractive index is deposited. A disperse layer of polystyrene nanoparticles is self-assembled on top of the dielectric film. The mean spacing of the particles is set by a combination of exposure time and ionic strength of the solution.** b** Optional step for shrinking the particles using UV/ozone etching (UVO) of the polystyrene.** c** A 10–20-nm-thick layer of Cr is deposited.** d** The particles are removed mechanically using tape stripping.** e** Reactive ion etching (RIE) with a high anisotropic etching rate for the dielectric layer and a negligible etching rate for Au and Cr is used to etch through the dielectric film at the exposed holes in the Cr mask.** f** The Cr mask is removed by wet etching

Although nanoporous waveguides were demonstrated using porous anodized aluminum, the published results indicate that a lower nanopore density over the large area of a waveguide would be beneficial. Hence, the design requirements for a suitable porous waveguide and for pore-spanning lipid membrane self-assembly are similar, i.e., a high refractive index oxide and low porosity are desirable. Traditional nanolithographic techniques, such as e-beam lithography, are not suitable for patterning nanostructures on unconventional substrates with large areas. However, alternative lithographic techniques built on self-assembly, such as colloidal lithography, make parallel nanopatterning of regular features at low cost possible [23, 24]. A platform on which such measurements could be combined was demonstrated for silicon nitride-based SPR-coupled waveguides [60]. Colloidal lithography with polystyrene beads [61] was used to define a thin Cr mask by physical vapor deposition and stripping of the polystyrene particles on the waveguide surface (Fig. 9a–d). The thin metal film is an excellent mask for reactive ion etching of straight pores through the silicon nitride film with the underlying Au film as the etch stop, and can easily be removed by a specific Cr wet etch (Fig. 9e–f).

**Fig. 10:**
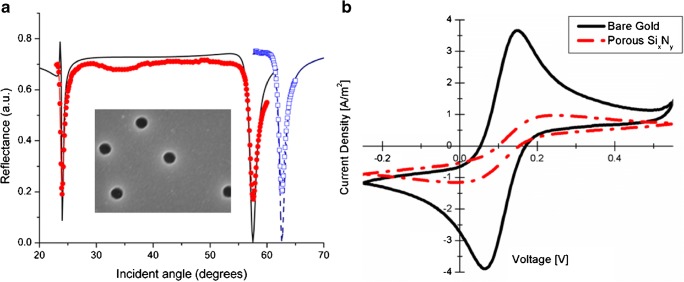
**a** Red circles are the angle spectrum of an SPR-coupled waveguide with 40 nm open pores (made using a mask of 100 nm colloids reduced in size to 40 nm, see electron micrograph inset) in air, using a p-polarized He–Ne laser. Blue open squares are for the same waveguide measured in water. Black lines are modeled data for the parameters of the waveguide [60].** b** Cyclic voltammogram showing the response of a pure macroscopic Au electrode in 2 mM K_4_Fe(CN)_6_ containing 100 mM KCl solution at 50 mV s^−1^ (solid black line) and for the nanoporous SiN_*x*_-coated Au electrode (dashed red line) under the same conditions. The etched porous film shows the S-shaped curve typical of a nanoelectrode [60]

Using colloidal lithography with a metal mask to define the pattern has several advantages over, e.g., e-beam lithography, on polymer resists. It is a parallel method that can be applied to the vast areas that are typically used for waveguide sensing. The metal mask allows for using reactive ion etching of high aspect ratio pores. The method also allows for independent tuning of the density, diameter, and depth of the pores in the waveguide films, i.e., using sufficiently thick waveguiding layer but still being able to etch a pore with sub-100 nm diameter through the entire film. However, it has the disadvantage that only short-range ordering of the nanopores can be achieved. Figure 10a shows an example of waveguide modes for an SPR-coupled silicon nitride waveguide sensor with nanopores defined by colloidal lithography [60]. Cyclic voltammetry using the underlying gold film as an electrode was shown to work simultaneously on the same sensor chip (Fig. 10b) [60]. Thus, all factors are in place for extending the tools of membrane biosensing and membrane biophysics research to the simultaneous measurement of membrane electrochemical properties, and kinetic processes of adsorption, binding, and conformational changes using a single waveguide chip.

### Polymer brush-functionalized nanoporous substrate as proton conductive membrane

Ionic transport across synthetic or natural barriers remains a matter of significant scientific curiosity. A range of important processes in nature are driven by ionic transport across biological membranes. ATP-sensitive potassium channels functioning as sensors for cell metabolism [62], transient receptor potential (TRP) cationic channels helping organisms in interpreting environmental stimuli [63], and G protein-gated inwardly rectifying potassium channel (GIRK) regulating neurotransmission [64, 65] exemplify the significance of ion channels that exist in nature. The precise and accurate operation of natural ion channels has inspired scientists from diverse fields of application and mimicking the function of natural channel in a completely synthetic arrangement remains an active area of research. Endeavors in this domain have led to the development of materials for a variety of applications including (bio)sensing, filtration, energy storage technologies, and energy generation technologies.

Advances in material science have produced a variety of ion-conducting channels bearing synthetic platforms derived from chemically diverse materials with a control over geometry and density of channels. Polymeric track-etched membranes, membranes fabricated from anodization of metals, ordered mesoporous thin films, porous silicon membranes produced by photoelectrochemical etching, membranes derived from electron beam lithography and ion beam sculpturing are representative examples of ion channels bearing synthetic platforms [66]. The ion conductivity and application domain of synthetic channels are synergistically related to their physical architecture and interfacial response stemming from the chemical identity of the surface functional groups. In addition, the extent of the interfacial response and the performance of synthetic ion channels bearing functional materials within a particular application domain can be finely modulated by decorating the surface of channels with an appropriate set of functionalities at a precisely controlled surface functional group density [67, 68]. In this context, surface-tethered assemblies of carefully designed macromolecules widely known as polymer brushes [69–71] have emerged as an effective avenue enabling precise control over the surface chemical functionality and functional group density. The emergence of hybrid materials derived from the combination of polymer brushes and synthetic ion channel platforms has played a key role in diversifying their respective application profile resulting in the development of a variety of functional hybrid materials. Capitalizing on this opportunity, we and others have developed a range of functional hybrid materials derived from synthetic ion channels and polymer brushes.

Employing a combination of polymeric track-etched membranes bearing an asymmetric conical nanochannel and zwitterionic polymer brushes, we have demonstrated an unprecedented example of fully synthetic nanochannel exhibiting pH-responsive permselectivity that could be switched between anionic/cation permselectivity, and finely tuned by controlling the pH of the environment [72]. A further fine tuning of cation permselectivity was achieved by tethering phosphoric acid groups bearing polyprotic polymer brushes on the surface of polymeric track-etched conical nanochannels [73]. In the same vein, we employed poly(vinyl pyridine)-based polymer brushes to fabricate a proton-gated cylindrical ion channel capable of mimicking biological ion channels and displaying a pH-responsive switching between “on” and “of” towards the transport of ions [74]. Mimicking the thermosensation that is a manifestation of thermal activation of temperature-sensitive ion channels distributed on the surface of sensory neurons [75, 76], our group has exploited the thermoresponsive actuation of poly(*N*-isopropyl acrylamide) polymer brushes and fabricated a polymeric track-etched conical nanochannel functioning as a thermally driven nanogate where opening and closing of the channel can be remotely controlled by tuning the temperature of the environment between 23 and 40 °C [77]. The potential of the combination of polymer brushes and porous materials for the development of smart ion channels has also been demonstrated using mesoporous silica membranes [78–81].

**Fig. 11:**
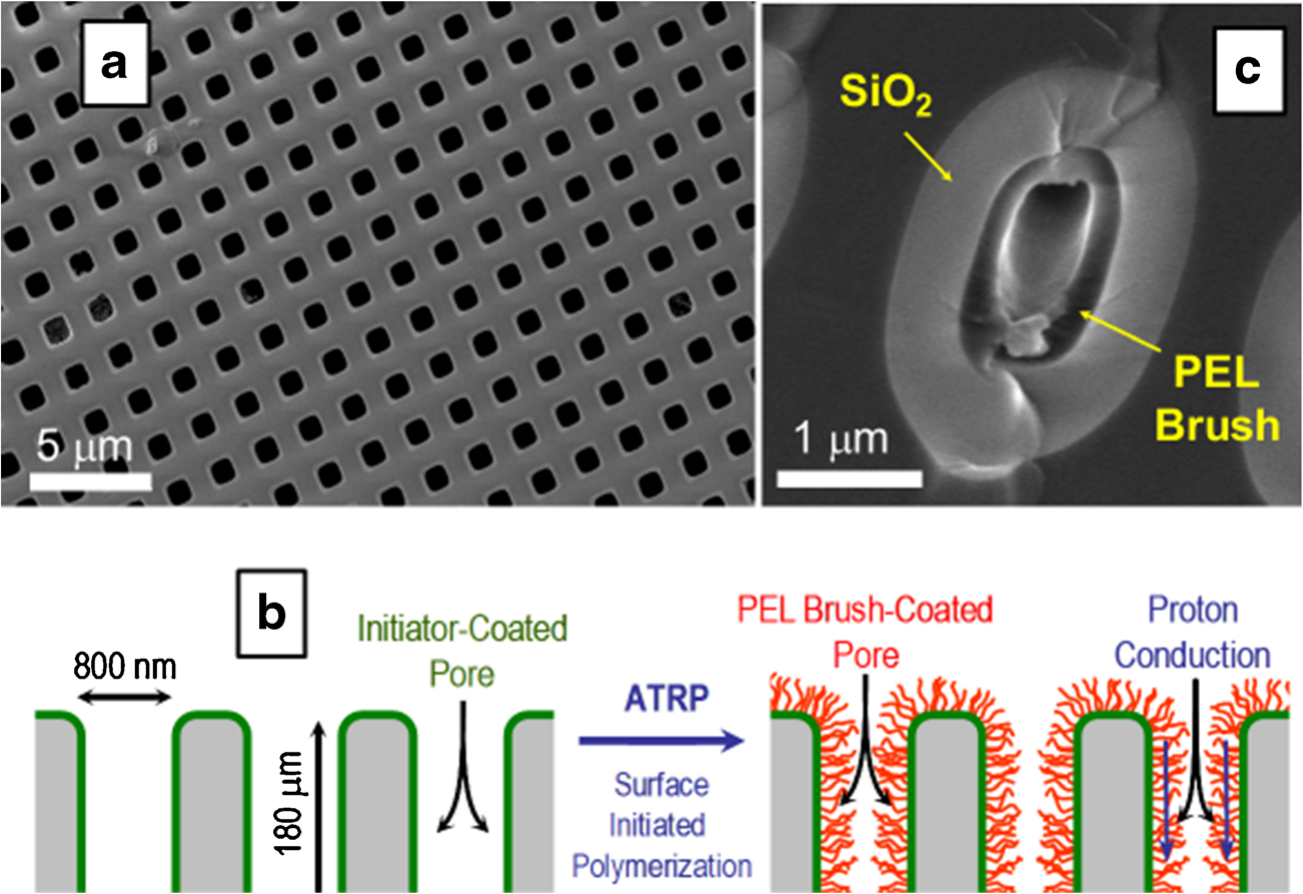
**a** SEM image showing the surface view of the highly ordered monodisperse vertically aligned channels of the macroporous silicon membrane.** b** Schematic illustration of the functionalization of channels of macroporous silicon membrane with polyelectrolyte brush.** c** SEM image showing the cross section of polyelectrolyte brush-functionalized channel of the macroporous silicon membrane. Adapted with permission from J. Am. Chem. Soc. 2008;130:13140–13144. Copyright (2008) American Chemical Society

**Fig. 12:**
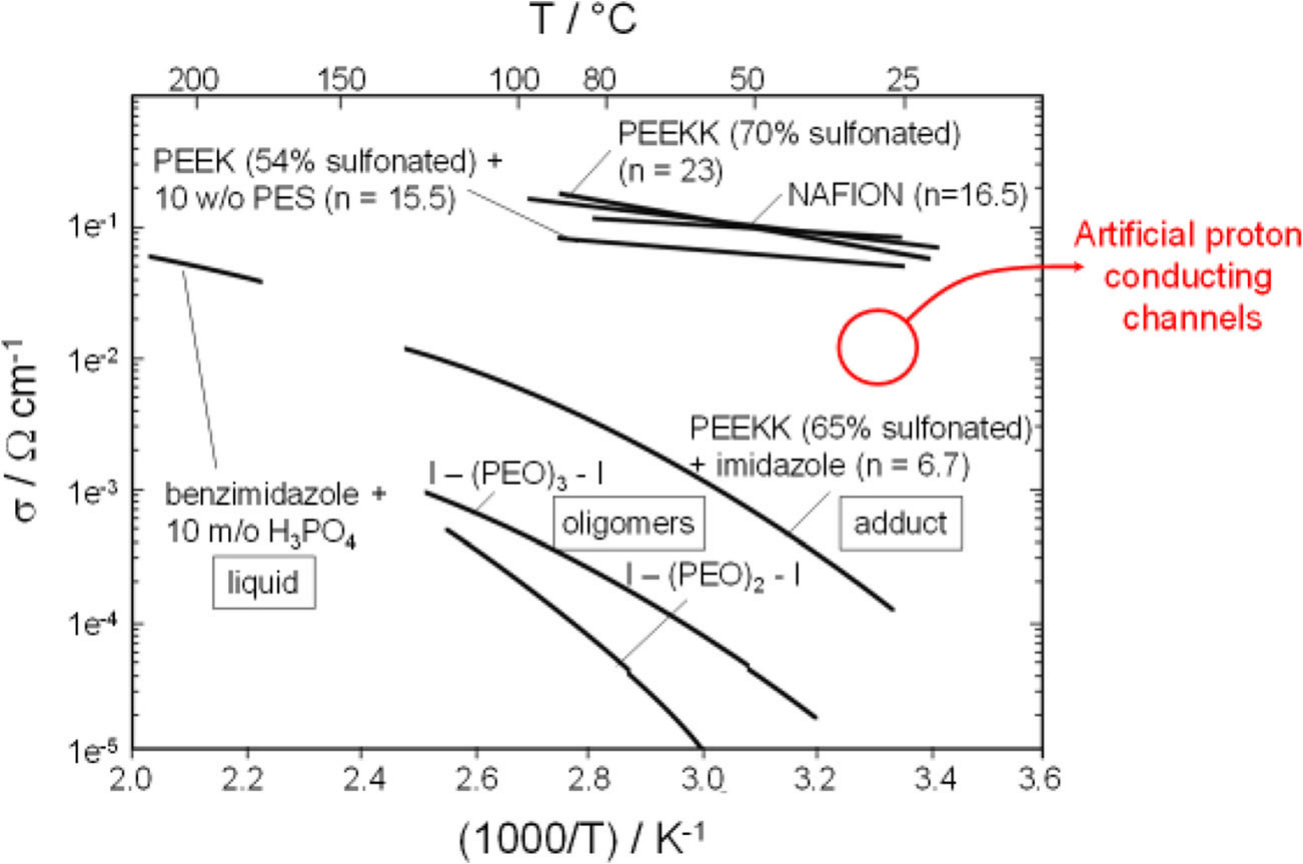
Overview of the proton conductivities of various materials that are being investigated for application as PEMs in fuel cells. Adapted with permission from J. Membr. Sci. 2001;185(1):29–39. Copyright (2001) Elsevier

Ion-conducting membranes are at the heart of electrochemical energy storage and conversion devices [82–89]. In fuel cells that are being developed as energy sources for moderate temperature applications, a proton-conducting membrane also referred to as proton exchange membrane (PEM) is necessary as a separator in electrode assembly and functions as an insulator for electrons and conductor for protons. The fuel such as hydrogen is oxidized at the anode to produce electrons and protons. The electrons are directed towards the outer circuit where they are used to perform useful work while the protons pass through the PEM and complete the electrochemical reaction by getting reduced to produce water as a by-product at the cathode. Nafion, a perfluorinated polyelectrolyte, is the most widely used PEM in fuel cells. The self-assembly of perfluorinated polyelectrolyte molecules of Nafion produces a membrane that consists of water filled nanoscopic channels that are lined by the sulfonic acid groups and embedded within the perfluorinated matrix of the Nafion. The protons are transported across the PEM through these water-filled nanoscopic channels as hydronium ions [89–92]. It is worth mentioning here that Nafion is considered as the gold standard despite experimental evidence that about 60% of the nanoscopic channels end blindly and do not contribute towards the proton transport [93]. The fluorinated nature of Nafion has been of serious environmental concern. In addition, concerns have been raised about the mechanical and chemical stability of Nafion, particularly when the fuel cell is operated at a moderately high temperatures [94]. These issues have triggered the search for alternates that could overcome the concerns associated with Nafion. In this context, we have proposed a simple yet effective approach to develop PEM membranes that exhibit superior proton-conducting behavior and help in overcoming the limitation associated with traditional PEM. Our approach relies on confining the proton-conducting moieties inside the highly ordered vertically aligned monodisperse channels [25, 95] of self-standing macroporous silicon membranes produced by the photoelectrochemical etching process. Surface initiated atom transfer radical polymerization (SI-ATRP) was employed to grow proton-conducting moieties (–SO_3_H) bearing poly(3-sulfopropylmethacrylate) (polySPM) polyelectrolyte (PEL) brushes inside the confined geometries of the macroporous silicon membrane (Fig. 11). The impedance spectroscopic analysis of this membrane revealed that the developed platform exhibited humidity-dependent proton-conducting behavior that is comparable to that of Nafion and exhibited a proton conductivity of 2 × 10^−2^ S/cm at 95% relative humidity (RH). The proton conductivity of this system was observed to increase from 7 to 15 mS/cm upon increasing temperature from 25 to 80 °C at 90 % RH. In a separate endeavor, we replaced potentially hydrolytically prone ester linkages bearing polySPM with a relatively stable amide bond bearing poly(2-acrylamide-2-methylpropane sulfonic acid) (polyAMPS) PEL brushes [96]. The resulting membrane exhibited proton-conducting behavior that was comparable to the membrane derived from polySPM. With successful demonstration of the effectiveness of our pore-filling approach for fabrication of proton-conducting channels, we focused our attention on a critical limitation of Nafion, i.e., high dependence of proton conductivity on RH [97]. The dehydration of nanoscopic channels of Nafion at lower humidity levels hampers its proton conductivity so severely that it essentially behaves as an insulator to protons. To overcome this challenge we evolved our molecular design and filled the channels of macroporous silicon membranes with a copolymer brush derived from a combination of the proton-carrying sulfonate groups bearing SPM monomer and a polyethylene glycol-based monomer monomethoxy oligo(ethylene glycol) methacrylate (MeOEGMA) as humidifying agent [98]. The introduction of humidifying agent was designed to enhance the ability of the membrane to achieve high levels of hydration even at lower levels of RH and reduce the dependency of the proton conductivity on relative humidity. The selection of polyethylene glycol-based monomer was triggered by its hydroscopic nature, which also makes its suitable as an additive for moisturizers and other cosmetic products. The ratio of the two monomers SPM/MeOEGMA in the final copolymer was found to be 0.87:0.13 and the membrane showed an ion exchange capacity (IEC) of 0.12 meq/g, which was about 14% lower than the IEC of the membrane produced by pore filling with the pure polySPM brush. The resulting platform exhibited high proton conductivity of about 10^−2^ S/cm over the entire range of RH from 30% to 90%. The proton conductivity of polySPM-*co*-MeOEGMA brush-filled macroporous silicon membrane was 4 × 10^−2^ S/cm at 30% RH which is several orders of magnitude higher than the proton conductivity of Nafion under identical conditions. In addition, the high proton conductivity of polySPM-*co*-MeOEGMA brush-filled macroporous silicon membrane remained constant when tested at different temperatures ranging from 20 to 100 °C at 90% RH. These traits clearly outperform the gold standard Nafion as well as a range of other PEMs being developed for applcation as proton-conducting membranes in fuel cells (Fig. 12). These results illustrate a promising strategy for fabricating tailorable proton-conducting membranes with highly optimized physical and chemical characteristics that could lead to new methods for the fabrication of PEMs.

### Polymer rod array waveguides by templating from AAO substrates

Nanoporous hard templates provide a two-dimensionally confined space in which self-organization processes of soft materials such as self-assembly, phase separation, and crystallization can be tuned easily [99, 100]. An advantage of hard templates is that they provide a range of space-related parameters (pore diameter, curvature, nature of pore walls) that can be used to induce or manipulate nanostructure morphology. Nowadays, a broad range of soft materials can be formed into nanotubes or solid nanorods by means of nanoporous hard templates containing arrays of well-ordered and well-aligned cylindrical nanostructures [101–104]. These templates are particularly suitable for the rational generation of mesoscopic fine structures in the form of nanorods because equilibrium and non-equilibrium states as well as a range of unprecedented confinement-induced morphologies with new and exciting properties can be realized [101, 105].

**Scheme 1:**
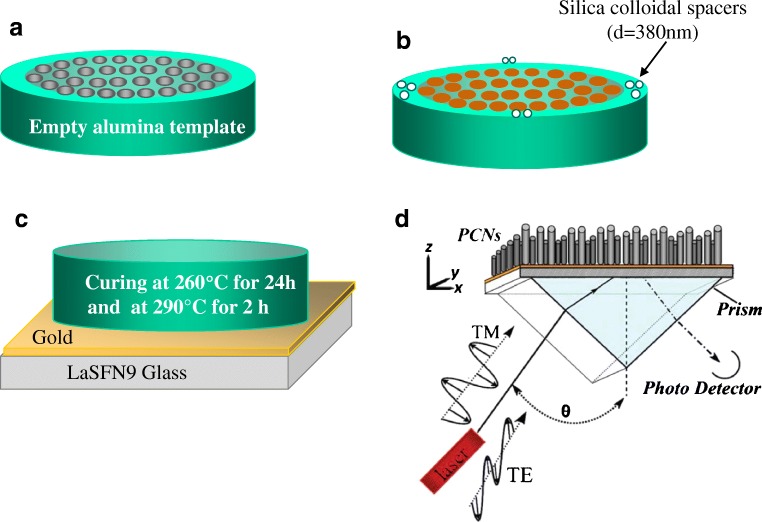
Fabrication of PCNs on Au surface. A 2-nm-thick Cr film and subsequently a 50-nm-thick Au layer were deposited on high refractive index glass (LaSFN9, Hellma Optik, *n* = 1.845). The Au surface was functionalized by immersion in 5 mM 2-aminoethanethiol in absolute ethanol solution.** a**,** b** CEMs infiltrated into the AAO (pore diameter 60 nm and 150 nm, pore depth 650 nm, lattice constant 105 nm) and kept under vacuum.** c** After removal of excess CEMs from the AAO surface, the CEMs-filled AAO was pressed against the gold-deposited glass substrates and thermally cured under N_2_.** d** The residual Al and AAO were then dissolved and an array of free-standing PCNs oriented normal to the substrate surface was finally obtained

**Fig. 13:**
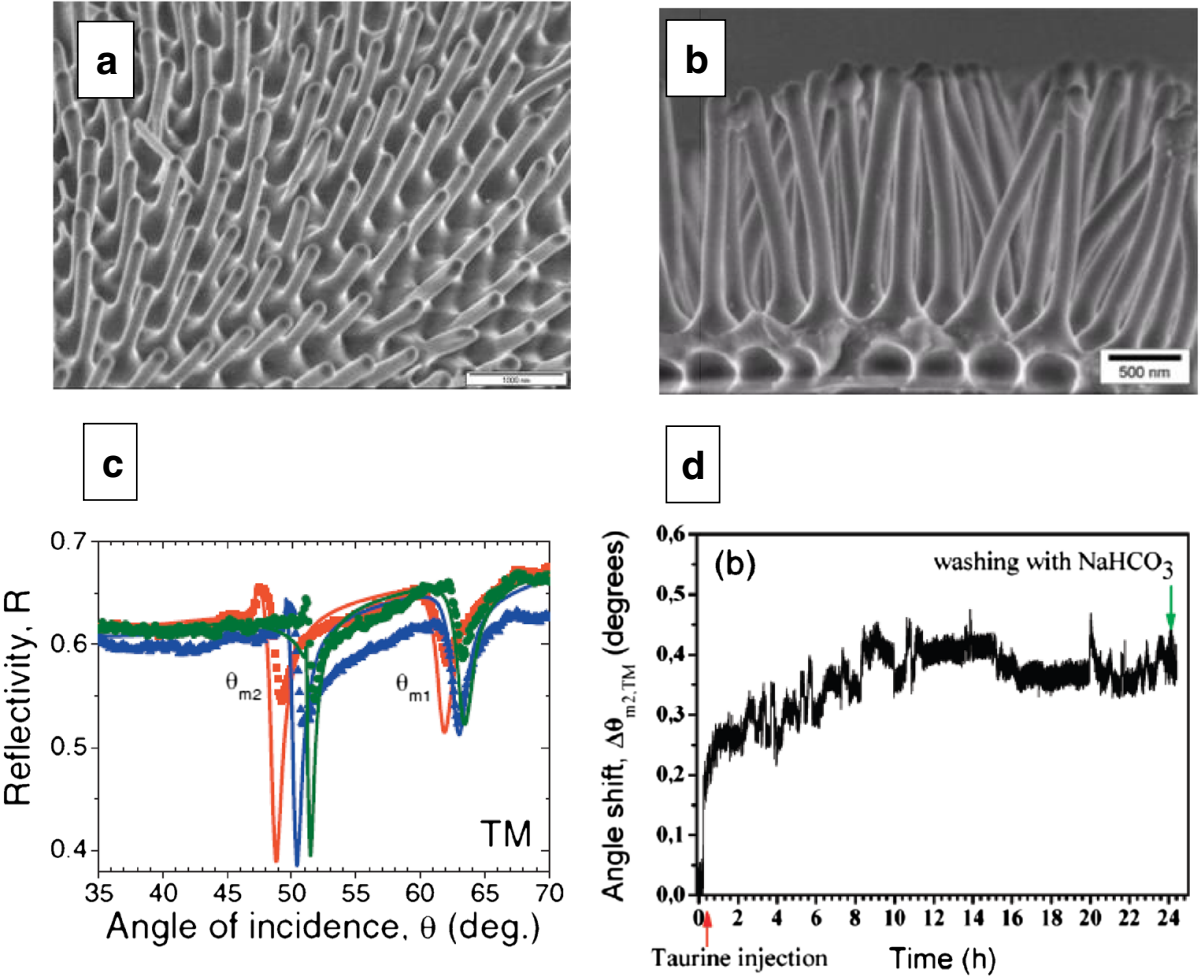
Top view **a** and side view **b** SEM image of PCNs with 150 nm diameter and 1.5 μm length situated on a Au-covered glass substrate. The nanorods are a replica of AAO nanopores with the same dimensions, wherein the polymerization took place.** c** Optical waveguide spectroscopy (OWS)* R* vs. θ scans of the PCN arrays in different environments in transverse magnetic mode: Milli-Q water (red squares); ethanol (blue triangles); isopropanol (green circles). PCN diameters are 60 nm and interrod distances are 105 nm.** d** Kinetics of taurine coupling to the PCNs tracked by changes in the angular shift of the second-order TM mode Δθ_*m*2_. Reprinted with permission from [113]. Copyright (2010) American Chemical Society

Nanotechnological applications of polymer nanorod arrays (PNAs) can be found in a great variety of fields, such as photovoltaic devices [106, 107], photonic crystal slabs [108, 109], electrochemical sensors [110], or biosensors [111–117]. Optical biosensing with PNAs has many advantages over that with porous oxide films (i.e., SiO_2_ or Al_2_O_3_) since these templates suffer poor stability in acid or alkali solutions, which might result in a deterioration of their sensing performance. Many times, a protective coating layer is usually applied on the inner walls of the porous membranes to enhance their pH stability [118]. In our previous work, we reported the template-assisted fabrication of polycyanurate thermoset nanorod arrays (PCNs) via thermal curing of cyanate ester monomers (CEMs) in AAO templates [113]. The fabrication of the PCNs is depicted in Scheme 1. CEMs were chosen owing to their (i) self-curing nature, (ii) low viscosity (liquid at room temperature) which facilitates ease of processing in nanomolding, (iii) high chemical and mechanical stability upon cross-linking, which gives sufficiently long lifetime, and most importantly (iv) residual cyanate groups that are available as reactive sites for further surface modification (i.e., attachment of biomolecules) [119]. As a result of dimensional optimization studies, it was observed that when the aspect ratio (*L*/*D*) of PCNs was below 11, they did not form bundles and they were mechanically more stable (Fig. 13a, b).

When fabricated on a Au surface, PCN arrays provide a platform for detecting molecular adsorption with sufficiently high sensitivity using optical waveguide spectroscopy (OWS) (Fig. 13c). We employed OWS to monitor surface modification of PCNs with a model molecule taurine. A PCN array was kept in aqueous 0.2 M NaHCO_3_ solution for 24 h to obtain a stable baseline prior to a* R* vs. θ scan revealing minimum reflectivity related to the second-order TM mode at an incident angle θ = 50.78°. Subsequently, 8 mM of taurine (*n* = 1.48) in 10 mL of 0.2 M NaHCO_3_ solution was injected at a rate of 0.5 mL/min. A plot of* R* against θ measured after taurine injection and washing with 0.2 M NaHCO_3_ solution (Fig. 13d) revealed that the reflectivity minimum associated with the second-order TM mode was shifted from θ = 50.78° to θ = 51.11°. The angular shift was unambiguously indicative of the attachment of taurine to the PCNs [113].

These studies proved that the inverse geometry of the AAO template, from a porous matrix to a “field” of parallel free-standing nanorods, practically retains the waveguiding capability, while providing further flexibility towards the functionalization of the polymer surface. In comparison to a planar waveguide, the high surface area of the PCNs provides increased sensitivity for biosensing, by amplifying the instrumental response, which is directly related to the amount of adsorbed material per unit area. The highest achievable figure of merit (FOM) was 196 reciprocal refractive index units (RIU^−1^) for the TM_2_ mode (*m* = 2). This value is almost three times higher than the previously reported value for regular angular modulation-based SPR sensors [120, 121].

Following these early studies, many research groups used nanorod or nanotube arrays made of organic or inorganic materials as optical waveguide-based biosensing applications [117, 122, 123]. They even reported improved limit of detection (LOD) and FOM results with these promising materials [117, 118, 122, 123].

**Fig. 14:**
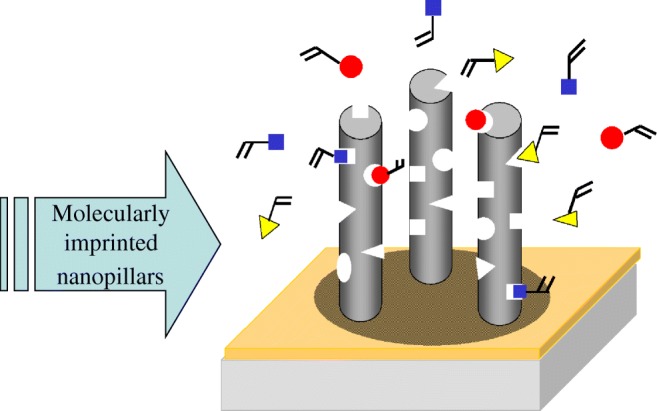
Extension of the nanorod array to a waveguide structure composed of molecularly imprinted polymer pillars for chemical and biomedical sensing

A very exciting extension of the nanorod array waveguide concept is sketched in Fig. 14. Firstly, the inner surfaces of the nanoporous membrane are functionalized with a template (analyte) molecule prior to the polymerization of the monomeric pore filling. In this way, almost all the template molecules are placed on the outermost surface of the nanorods after dissolution of the matrix structure. As a result of the proximity of the template molecules to the outer surface, the distribution of effective binding sites on imprinted nanoparticles after template extraction is considerably improved. This new structure is called molecularly imprinted polymeric rod array (MIP-NRAs) and offers a number of advances over planar arrangements [124], but also compared to MIP nanoparticles coupled to a hydrogel waveguide [125]: (1) the inner surface area of this structure is much higher than the geometrical dimensions of the planar waveguide; (2) analyte solution can freely diffuse through the MIP nanorods; (3) the optical interrogation of the polymeric cavities, filled with the analyte molecules, occurs throughout the cross section of the waveguide (and not just on its surface).

## Conclusions

Nanoporous thin films as optical waveguides offer very attractive diagnostic approaches for the label-free detection of analyte molecules of very different chemical nature, size, shape, and other physical properties. We presented waveguides from a range of different materials, fabrication protocols, and applications in diverse fields of research and development with the intent to demonstrate their versatile use. With the ever-increasing demand for label-free detection of marker molecules in medicine and biotechnological applications, for the monitoring of surface processes like analyte binding, for the analysis of complex supramolecular architectures like lipid bilayer membranes, or for the quantitative assessment of interfacial processes, e.g., in fuel cell membranes, the guidance of optical modes in these thin-layer structures still offers an impressive space for further contributions to the development of direct optical sensing concepts for analytical and bioanalytical applications.
